# Study of the accuracy of a radial arterial pressure waveform cardiac output measurement device after cardiac surgery

**DOI:** 10.1186/s13019-023-02128-1

**Published:** 2023-01-17

**Authors:** Pilar Ordoñez-Rufat, Nuria Mancho-Fora, Cristian Tebe-Cordomi, Victoria Polit-Martinez, Ricardo Abellan-Lencina, Joaquin Fernandez-Alvarez, Juan Carlos Lopez-Delgado

**Affiliations:** 1 Intensive Care Department, Bellvitge University Hospital, C/ Feixa Llarga s/n, 08907 Hospitalet de Llobregat, Barcelona Spain; 2 Biostatistics Department, Universitat de Barcelona, Campus Bellvitge, Av. Mare de Déu de Bellvitge, 3, 08907 Hospitalet de Llobregat, Barcelona Spain; 3 IDIBELL (Biomedical Research Institute of Bellvitge), Avda. de La Granvia de L’Hospitalet, 199, 08908 Hospitalet de Llobregat, Barcelona Spain

**Keywords:** Cardiac surgery, Pulmonary artery catheter, Arterial pulse contour analysis, Hemodynamic monitoring

## Abstract

**Background:**

Less invasive monitoring, such as radial arterial pulse contour analysis (ProAQT® sensor), represents an alternative when hemodynamic monitoring is necessary to guide postoperative management and invasive monitoring is not technically feasible. The aim of the study is to evaluate the accuracy of the ProAQT® sensor cardiac output measurements in comparison with Pulmonary Artery Catheter (PAC) during the postoperative course of patients who underwent cardiac surgery with cardiopulmonary bypass.

**Case presentation:**

Prospective observational study in a Surgical Intensive Care Unit of a tertiary university hospital. Ten patients with a mean age of 73.5 years were included. The main comorbidities were hypertension, diabetes, dyslipidemia and the preoperative left ejection fraction was 43.8 ± 14.5%. Regarding the type of surgery, six patients underwent valve surgery, two underwent coronary artery bypass grafting and two underwent aortic surgery. The cardiac index measured simultaneously by the ProAQT® sensor was compared with the PAC. The parameters were evaluated at predefined time points during the early postoperative courses (6 h, 12 h, 24 h, 48 h and 72 h). The degree of agreement with the cardiac index between the PAC and the ProAQT® sensor along the time points was measured using the concordance correlation coefficient, Bland–Altman analysis, and four-quadrant plot. Sixty-three pairs of measurements were analyzed. We showed that measurements of cardiac index were slightly higher with PAC (β ^ = − 0.146, *p*-value = 0.094). The concordance correlation coefficient for the additive model of cardiac index was 0.64 (95% Confidence Interval: 0.36, 0.82), indicating a high concordance between both sensors. Bland-Altmann analysis showed a mean bias of 0.45 L·min^−1^·m^−2^, limits of agreement from − 1.65 to 2.3 L·min^−1^·m^−2^, and percentage of error was 82.5%. Four-quadrant plot of changes in cardiac index showed a good concordance rate (75%), which increases after applying the exclusion zone (87%).

**Conclusions:**

In patients undergoing cardiac surgery, the ProAQT® sensor may be useful to monitor cardiac index during the postoperative period, especially when more invasive monitoring is not possible.

**Supplementary Information:**

The online version contains supplementary material available at 10.1186/s13019-023-02128-1.

## Background

The perioperative course of cardiac surgery is associated with rapid hemodynamic variations due to bleeding, low cardiac output and vasoplegia. Monitor hemodynamic parameters, such as cardiac index (CI), are helpful for guiding and optimizing fluid resuscitation and response to drug administration (i.e., vasopressors and inotropes), which is crucial in the early stages of the postoperative period to achieve adequate tissue perfusion, and it may ultimately improve outcomes [1].

The pulmonary artery catheter (PAC) remains the gold standard for hemodynamic monitoring, and it is the only device for which there is a high level of evidence and safety [2]. However, PAC represents an invasive procedure that is not always practicable in cardiac surgery (i.e., tricuspid valve surgery and left bundle branch block are relative contraindications). In addition, it cannot measure dynamic parameters (e.g., systolic volume variation) [3].

The use of PAC should be restricted for the most severely ill (e.g., refractory shock and right ventricular failure) and less invasive monitoring devices may be more appropriate for guiding fluid resuscitation [2, 3]. The hemodynamic monitoring is based on theoretical concepts that have been validated through PAC, and several devices based on waveform analysis have been developed. Among such devices, the ProAQT® sensor and Pulsioflex® monitoring platform (Pulsion Medical Systems SE., Munich, Germany) provide both a radial arterial pulse contour analysis similar to the Pulse Index Continuous Cardiac Output (PiCCO2) device with the advantage of being even less invasive [4]. In addition, it proves an automatic calibration without the need of manual transpulmonary thermodilution [5]. The accuracy of the parameters measured by this device has been compared with PiCCO2, but only one study has compared the ProAQT® sensor with PAC in cardiac surgery patients to date [6]. We hypothesized that CI from the ProAQT® sensor could be compared with PAC as gold standard in hemodynamic monitoring when this is possible, to validate those parameters and their usefulness. The main aim of the present study was to compare CI in both PAC and radial arterial pulse variation catheter (ProAQT®) to evaluate the accuracy of radial arterial pressure waveform CI measurement during the postoperative period of cardiac surgery.

## Case presentation

### Methods

A prospective observational study was performed in a surgical Intensive Care Unit (ICU) of a university affiliated referral hospital between June 2017 and September 2018. Patients scheduled for elective cardiac surgery were studied prospectively. We included those patients in whom PAC (Swan-Ganz®, Edwards Lifesciences Co., Irvine, California, USA) was indicated by the attending physician on admission after cardiac surgery. The main reason for PAC insertion was low cardiac output syndrome (LCOS) in all cases. We excluded patients with active bleeding, especially those leading to a shock status, chronic atrial fibrillation, need of Intra-Aortic Balloon Pump, surgical causes of hypotension (e.g., cardiac tamponade) and emergency surgery. Based on manufacturer’s instructions, the ProAQT® sensor is less reliable under these clinical circumstances, especially in the presence of unresponsive hemodynamic instability. A flow chart of the studied patients is showed in Additional file 1: Fig. S1.

The study was approved (approval number 347/15) by the Institutional Ethics Committee of our hospital (Comitè d’Ètica i Assajos Clínics de Hospital Universitari de Bellvitge; Barcelona, Spain) and informed consent was obtained from all individual participants in the study.

Data was prospectively extracted from the medical registry of each patient and collected in a local database by the investigators in real time for analysis purposes. We registered both PAC and radial arterial pulse variation catheter (ProAQT®) parameters simultaneously.

All patients were monitored continuously by means of pulse oximetry, invasive mean arterial pressure (MAP), continuous ECG and temperature monitoring. A radial artery catheter and a central venous catheter were inserted before surgery. The PAC (Swan Ganz®, Edwards Lifesciences) was inserted via the right internal jugular or the left subclavian vein and was connected to a Vigilance II® monitor (Edwards Lifesciences) to obtain continuous CI monitoring by means of an automated continuous pulmonary arterial thermodilution-derived cardiac index. A thermal filament on the PAC detects changes in blood temperature to calculate CI throughout a modified Stewart-Hamilton equation. The correct position of the PAC was confirmed using pressure curve waveform and a chest-X ray.

Once PAC was inserted, a radial artery catheter was connected to a ProAQT® sensor, which was plugged to the Pulsioflex® monitor (Pulsion Medical System, Munich, Germany). We followed the start algorithm based on biometric values (i.e., gender, age, weight, and height) with an automated calibration system. The CI is then calculated beat-to-beat by pulse contour analysis.

Hemodynamic data was recorded on admission, at 6 h, 12 h, 24 h, 48 h and 72 h with a 15 min window (e.g., the both measurements were performed at 6 h ± 15 min) at the same time for both devices. We performed additional measurements at 96 h (day 4) in two patients and at 120 h (day 5) in one patient. All measurements were performed under controlled invasive mechanical ventilation to avoid the influence of higher respiratory workload and higher variations of transpulmonary pressure on our data. Total numbers of measurements were 63. At all sets of measurements, we performed an auto-calibration of the Pulsioflex® and we flushed and zeroed pressure lines to avoid under or overdamping of a line. No measurement was performed during a fluid challenge.

Regarding invasive mechanical ventilation, tidal volume was between 6 and 8 mL·Kg^−1^ (ideal body weight), positive expiratory pressure was set between 3 and 6 cmH_2_O, fraction of inspired oxygen was adjusted to maintain oxygen saturation > 94%, respiratory frequency was adapted for an end-tidal carbon dioxide value between 35 and 40 mmHg. A Remifentanil infusion was titrated for an appropriate sedation according to patient responsiveness.

The surgical procedure was performed by the same group of surgeons during the study period following standards of practice. Priming volume of the circuit was between 500 and 800 mL. In all patients, decisions regarding perioperative management were made by the attending physician according to local protocols. Patients were treated according to hemodynamic parameters and metabolic markers of tissue perfusion, such as arterial lactate levels and venous oxygen saturation. Fluid resuscitation was performed based on local protocol following a restricted fluid regimen to avoid excessive positive fluid balance (i.e., < 2 L of positive fluid balance per day) [7]. Our hemodynamic objectives were to achieve both appropriate mean arterial pressure (MAP) (i.e., about 60–70 mmHg or the previously reported usual MAP in each patient), urine output (i.e., > 0.5 mL·kg^−1^·h^−1^ or higher to avoid positive fluid balance) and appropriate CI (about 2.2–2.5 L·min^−1^·m^−2^) [8]. We monitored central venous pressure (CVP) to see the CVP changes over time during ICU admission. We evaluated passive leg raise for hemodynamic management (i.e., fluid responsiveness) and fluid loading was performed by crystalloids. According with our previous research, our perioperative transfusion trigger was a hemoglobin value between 7 and 8 g·dL^−1^ [9]. Fluid therapy was guided by ICU specialist based on PAC parameters and continuous hemodynamic parameters (i.e., MAP, central venous pressure, heart rate and urine output). It is important to note that the attending physician was blinded for Pulsioflex® monitor data.

### Statistical analysis

Categorical variables were described by frequencies and proportions. Continuous demographic and clinical characteristics were either summarized through means and standard deviations or with medians and first and third quartiles if they were non-normally distributed. Normal distribution was assessed using Shapiro-Wilks test and quantile plots.

The degree of agreement with CI between PAC and the ProAQT® sensor across time points and participants was measured by the concordance correlation coefficient applied for repeated measures, expressed in terms of the variance components of a linear mixed model. Two models were estimated using CI as the responses variables and the type of sensor and time as explanatory variables, interaction term between type of sensor and time was also assessed [10]. Analyses were accompanied with the Bland–Altman plots for CI. Additionally, we also used the four-quadrant plots approach to investigate the ability of the ProAQT® device to detect serial changes in CI (i.e., trending ability), an exclusion zone for each four-quadrant plots were selected on the basis of current recommendations and previous literature, which represents 15% of the mean CI [11]. It is important to highlight that there is a lack of well-defined cutoff values for good, acceptable, and poor trending ability based on concordance rate in scientific literature [12]. All statistical analyses were performed using R (version 3.4.4) and statistical significance was established through α < 0.05 criterion. The statisticians were blinded for the type of sensor or device.

### Results

Ten patients were recruited for the study with a median age of 73.5 (Q1 = 68, Q3 = 76.5) years and all of them were male. The main comorbidities were hypertension (n = 8), diabetes (n = 5), dyslipidemia (n = 9) and preoperative left ejection fraction was 43.8 ± 14.5%. Two of them suffer from a mild degree of Chronic Obstructive Pulmonary Disease. Mean body mass index was 29.5 ± 6.5 kg·cm^−2^. Regarding type of surgery, six patients underwent valvular surgery (five mitral and one aortic), two coronary artery bypasses, and two aortic surgeries. Cardiopulmonary bypass (CPB) duration was 119 (Q1 = 91, Q3 = 182) and aortic cross-clamp time was 94 ± 51 min. All patients were under vasopressors (i.e., noradrenaline) and inotropes (i.e., dobutamine), and all of them were under mechanical ventilation when measurements were performed. Two patients suffered from atrial fibrillation postoperatively, but none of the measurements were performed under atrial fibrillation. Four of them experienced Type I of Acute Kidney Injury, without any influence over fluid balance. Our patients needed invasive mechanical ventilation for a prolonged time in comparison (49 ± 35 h) to uncomplicated patients, with reduced arterial partial pressure of O2 and fraction of inspired oxygen (PaO2/FiO2) ratio (148 ± 36). None of them died and the whole cohort was discharged from the hospital, with a mean ICU and hospital length of stay of 17 ± 9 and 25 ± 14 days respectively. Two patients were discharged to a rehabilitation facility. Clinical characteristics and main postoperative complications of the patients are summarized in Additional file 1: Table S1.

**Fig. 1:**
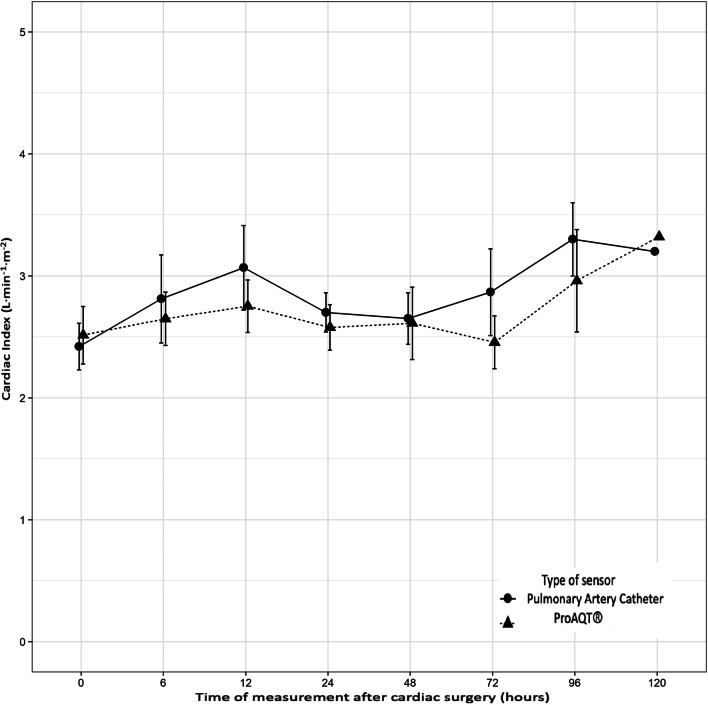
Comparison of mean cardiac index (with 95% confidence interval) measurements with pulmonary arterial catheter and ProAQT® sensor

Table 1 summarizes the hemodynamic data and inotropic/vasopressor needs measured during ICU stay. Despite measurements of CI were slightly higher with PAC sensor, we did not find any statistical difference in mean measurements of both CI (Fig. 1). The CI did not show any significant interaction between the type of sensor and time (Table 2). Although measures form ProAQT® were slightly lower and approaching statistical significance (β ^ = − 0.146, *p*-value = 0.094), the concordance correlation coefficient for the additive model was 0.64 (95% Confidence Interval: 0.36, 0.82), indicating a high concordance between the Swan-Ganz® catheter and ProAQT® sensor.

**Table 1:** Descriptive hemodynamic parameters during post-operative course of ICU admission

	ICU admission	6 h	12 h	24 h	48 h	72 h
HR (pbm)	98 ± 9	97 ± 9	97 ± 7	102 ± 6	93 ± 10	96 ± 9
SBP (mmHg)	125 ± 14	134 ± 19	132 ± 19	128 ± 24	131 ± 17	127 ± 21
DBP (mmHg)	62 ± 12	60 ± 10	60 ± 8	58 ± 10	57 ± 8	57 ± 11
MAP (mmHg)	82 ± 11	84 ± 10	83 ± 10	82 ± 13	81 ± 8	80 ± 13
CVP (mmHg)	13 ± 3.7	15 ± 4.5	14 ± 3	13 ± 4	15 ± 3	15 ± 5
Pulmonary artery catheterization data (Swan Ganz®)						
CI (L·min^−1^·m^−2^)	2.5 ± 0.7	2.7 ± 1.1	2.9 ± 0.7	2.9 ± 1.3	2.7 ± 0.2	3.1 ± 0.8
SPP (mmHg)	38 ± 10	39 ± 10	39 ± 12	38 ± 10	45 (37, 52)	47 ± 10
DPP(mmHg)	20 ± 5	20 ± 5	21 ± 7	22 ± 6	22 ± 5	21 ± 3
PAMP (mmHg)	26 ± 6	26 ± 6	27 ± 8	27 ± 7	29 ± 7	31 ± 5
PAOP (mmHg)	15 ± 4.4	16 ± 4	17 ± 5	20 ± 6	17 ± 5	17 ± 6
SVRI (dyn·s^−1^·cm^−5^)	1854 ± 777	2047 ± 467	1944 ± 721	1863 ± 503	1808 ± 349	1708 ± 576
PVRI (dyn·s^−1^·cm^−5^)	330 ± 139	282 ± 171	543 ± 159	359 ± 105	385 ± 140	381 ± 143
Radial arterial pulse variation catheter data (ProAQT®)						
CI (L·min^−1^·m^−2^)	2.4 ± 0.6	2.7 ± 0.6	2.6 ± 0.4	2.6 ± 0.7	2.6 ± 0.8	2.5 ± 0.5
SVV (%)	17 ± 7	17 ± 8.7	16 ± 6	17 ± 7	18 ± 9	17 ± 3
PVV (%)	13 ± 7.6	13 ± 7.7	14 ± 5	17 ± 8	13 ± 5	14 ± 5
SVRI (dyn·s^−1^·cm^−5^)	1950 ± 787	2125 ± 414	1859 ± 451	1907 ± 471	2245 ± 502	2177 ± 598
Inotropic and vasopressor needs						
NA (µg·Kg^−1^·min^−1^)	0.12 ± 0.05	0.11 ± 0.03	0.12 ± 0.06	0.11 ± 0.03	0.08 ± 0.07	0.07 ± 0.04
DBT (µg·Kg^−1^·min^−1^)	8 ± 6	9 ± 5	9 ± 5	8 ± 6	7 ± 5	7 ± 4

Values are presented as mean ± standard deviation

*HR* hear rate, *SBP* systolic blood pressure, *DBP* diastolic blood pressure, *MAP* mean arterial pressure, *CVP* central venous pressure, *CI* cardiac index, *SPP* systolic pulmonary pressure, *DPP* diastolic pulmonary pressure, *PAMP* pulmonary arterial mean pressure, *PAOP* pulmonary artery occlusion pressure, *SVRI* systemic vascular resistance index, *PVRI* pulmonary vascular resistance index, *SVV* stroke volume variation, *PPV* pulse pressure variation, *NA* noradrenaline, *DBT* dobutamine

**Table 2:** Linear mixed model for cardiac index

	Model 1					Model 2				
	Value	SE	DF	t-value	p-value	Value	SE	DF	t-value	p-value
(Intercept)	2.540	0.214	91	11.866	0.000	2.420	0.232	84	10.423	0.000
Sensor2	− 0.146	0.086	91	− 1.694	0.094	0.094	0.198	84	0.474	0.637
t6	0.238	0.179	91	1.332	0.186	0.367	0.229	84	1.601	0.113
t12	0.398	0.181	91	2.198	0.030	0.622	0.229	84	2.716	0.008
t24	0.171	0.173	91	0.991	0.324	0.280	0.222	84	1.260	0.211
t48	0.115	0.186	91	0.622	0.535	0.182	0.237	84	0.768	0.445
t72	0.358	0.205	91	1.749	0.084	0.612	0.260	84	2.352	0.021
t96	0.457	0.317	91	1.443	0.152	0.672	0.398	84	1.689	0.095
t120	0.171	0.429	91	0.398	0.692	0.156	0.540	84	0.289	0.774
Sensor2:t6						− 0.257	0.288	84	− 0.893	0.375
Sensor2:t12						− 0.468	0.294	84	− 1.591	0.115
Sensor2:t24						− 0.217	0.281	84	− 0.773	0.442
Sensor2:t48						− 0.133	0.298	84	− 0.446	0.657
Sensor2:t72						− 0.506	0.324	84	− 1.561	0.122
Sensor2:t96						− 0.434	0.486	84	− 0.893	0.374
Sensor2:t120						0.026	0.658	84	0.040	0.969

*SE* standard error, *DF* degrees of freedom. Sensor 2 corresponds to ProAQT® sensor

Model 1 (Additive Model): AIC (Akaike information criterion) = 207.53, BIC (Bayesian information criterion) = 241.4, ICC (intraclass correlation coefficient) = 0.81. Model 2 (Interactive Model): AIC = 220.56, BIC = 271.22, ICC = 0.78

**Fig. 2:**
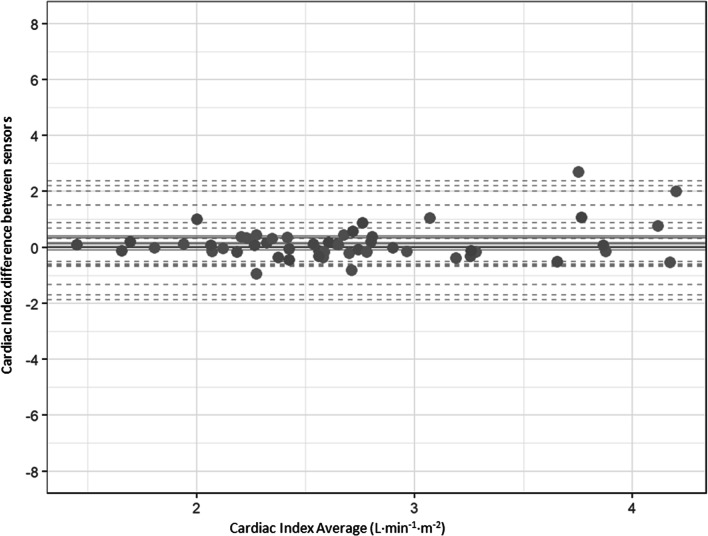
Bland-Altmann plot between cardiac index obtained with pulmonary arterial catheter and cardiac index obtained using ProAQT® sensor

Bland-Altmann analysis between CI measured with PAC and CI measured with ProAQT® sensor showed a mean bias of 0.45 L·min^−1^·m^−2^ and limits of agreement from − 1.65 to 2.3 L·min^−1^·m^−2^. When repeated measurements were considered, mean bias was 0.45 L·min^−1^·m^−2^ and 95% limit of agreement ranged from − 1.75 to 2.5 L·min^−1^·m^−2^ (Fig. 2). The percentage of error was 82.5%.

**Fig. 3:**
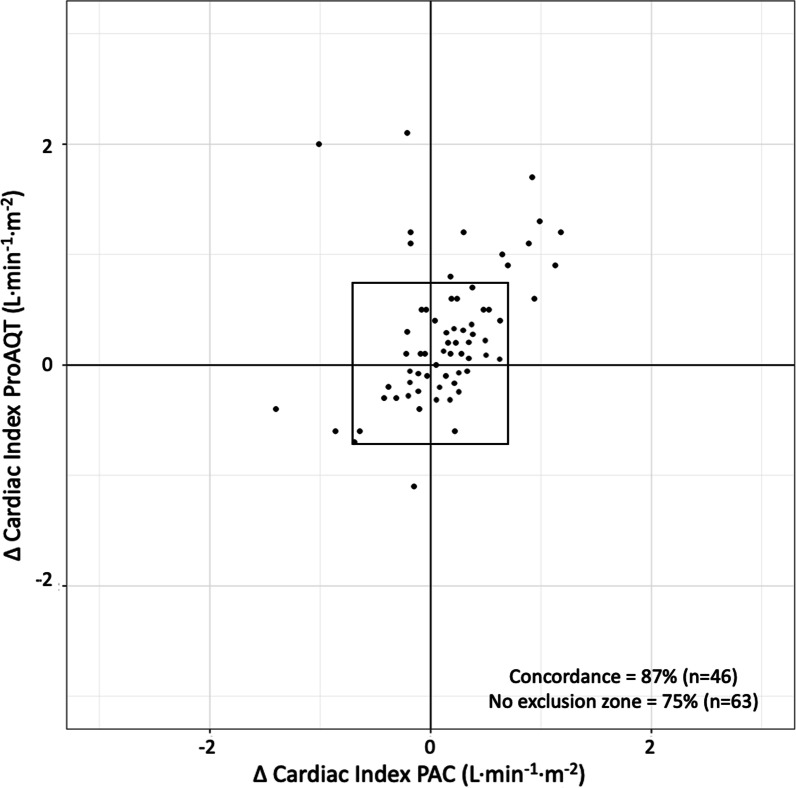
Four quadrant plots of changes in cardiac index measured with the ProAQT® sensor against the changes in cardiac index measured with pulmonary arterial catheter

Figure 3 shows the four-quadrant plot of changes in CI measured with the ProAQT® sensor against the changes in CI measured with PAC. The serial changes in CI measured with the ProAQT® were plotted against the changes in CI measured by PAC. The four-quadrant concordance rate, defined as the percentage of the number of data points that fall into 1 of the 2 quadrants of agreement are shown, with and without making use of an exclusion zone of 0.5 L·min^−1^·m^−2^ based on mean CI. The correlation coefficient without the exclusion zone was 0.30 (*P* < 0.001) and the concordance rate was 75%, which increases to 87% after applying the exclusion zone.

Systemic Vascular Resistance Index (SVRI), which is obtained in both sensors, showed slightly higher values when derived from ProAQT® (see Table 1). In consequence, we performed an extra analysis similar to CI with SVRI data. Our results suggest moderate concordance rates regarding SVRI derived from ProAQT® when compared with those derived from PAC (see Additional file 1: Table S2 and Fig. S1).

## Discussion and conclusions

Based on our results, the present study may suggest that CI measurements obtained by means of calibrated radial arterial pulse variation catheter (ProAQT®) may be similar from those obtained with the PAC after complicated major cardiac surgery. To our knowledge, this is the first study that has evaluated the accuracy of ProAQT® sensor measurements in comparison with PAC during the postoperative course of patients who underwent cardiac surgery with CPB. A similar study was performed but in patients who underwent off-pump bypass surgery [6]. The studied population represents complicated cardiac surgery patients requiring respiratory and hemodynamic support during immediate postoperative period, which it was the main reason to perform PAC insertion. It is important to remark that our results did not reflect the initial vasoplegia (i.e., low resistance and low cardiac index) thanks to the good hemodynamic response to therapy (i.e., fluids and vasopressors) of patients that we included in the study.

We agree that the use of PAC should be ideally restricted to the most complex hemodynamic scenarios whereas less-invasive monitoring, such as ProAQT® sensor, should be used in more stable patients [2, 13]. Hemodynamic monitoring is necessary in unstable patients after cardiac surgery for guiding fluid resuscitation, and the use of less-invasive devices is appropriate when there is a contraindication for the use of PAC or more invasive devices [1, 2]. Despite the lack of accuracy compared with the gold standard, some clinical conditions (e.g., inability to monitor the femoral artery for PiCCO2 placement) make impossible the use of invasive monitoring and they leave less invasive devices as the only choice for any type of hemodynamic monitoring. In addition, the ProAQT® sensor has the advantage of not needing an additional line placement or procedure since a radial artery is used to be placed in each patient who underwent cardiac surgery.

Similar to previous studies, our measurements obtained by an arterial pressure waveform sensor have a limited accuracy, which may be inherent to the technology of these less-invasive devices [4–6]. To improve accuracy of measurement as much as possible, we have used only auto-calibrated mode in the ProAQT® sensor since it seems to improve measurements, especially the trending ability of CI [5]. Indeed, the limited precision of uncalibrated measurements of CI obtained by less invasive devices has been widely reported in surgical patients [14].

It is important to point out that the technology is based on algorithms incorporating data on normal vascular anatomy and function, which is not the case of almost every patient [14]. The absolute values of CI measured by ProAQT® sensor after cardiac surgery has been shown to be reliable whereas a high percentage error has been reported in shock patients admitted to the ICU [6, 15]. Thus, our results of CI are moderately accurate, especially if we consider we have performed an evaluation in a short sample of complicated cardiac surgery patients.

The ProAQT® sensor measurements are based on arterial pressure waveform analysis and a severe vasoplegia, which is reflected by low SVRI, can influence the accuracy of these measurements [16]. It has been shown that the inaccuracy of CI measurements in ProAQT® sensors may be related with large variations of SVRI during major surgery (i.e., liver transplantations) [4]. However, vasopressor use may help to correct these variations and the influence of SVRI over CI could be minimal [16]. In addition, these variations have been reported during surgery and not during postoperative period, which could be largely influenced by hypovolemia caused by intraoperative bleeding and insensitive losses, especially in major abdominal surgeries. Despite our patients representing a complicated cohort of postoperative cardiac surgery patients needing vasopressor and inotropic support; we think that vasoplegia might have slightly influenced our results regarding SVRI since patients showed appropriate MAP and urine output during the study period.

Our patients also showed a prolonged ventilation post-surgery, probably due to two related factors: the hemodynamic and respiratory function of the patients. Postoperative pulmonary dysfunction is not frequent after cardiac surgery, but prolonged CPB time and low cardiac output syndrome are associated with prolonged mechanical ventilation [1]. In addition, patients showed low initial PaO2/FiO2 ratio which is associated with prolonged mechanical ventilation [2].

Our study presents certain limitations. The most important are the single-centre observational nature of our study and the lower size of our sample. Despite our results should be taken cautiously, the methodology we have used to evaluate measurements of ProAQT® sensors with PAC seems appropriate [10–12, 14]. Another point of criticism could be the measurement of CI by means of continuous thermodilution instead of intermittent thermodilution, which has been considered clinical gold standard. However, continuous thermodilution monitoring of CI with PAC has proven to be accurate and precise in the critically ill patients when compared with the "standard" intermittent bolus thermodilution technique, even when hemodynamics are highly variable (e.g., during cardiac surgery interventions) [17, 18]. Indeed, bolus thermodilution CO measurements may be affected by variations in injectate volume, rate, and temperature. Recovering from hypothermia after CPB may affect bolus thermodilution CO measurements until achieving normal temperature. These variations are eliminated when CI is measured by a continuous automated thermal technique, which has been performed in our study [19, 20]. Finally, our study showed a potential for selection bias and slow recruitment. As we previously remark, we included patients with good response to therapy, and as a result ProAQT® sensor measurements are as reliable as possible since they did not have any limitation (e.g., atrial fibrillation) that ProAQT® entails.

In our opinion, a task force guided by scientific societies involving all the healthcare professionals involved in hemodynamic monitoring should establish the standard conditions for the design and development of validation studies for these types of devices.

In conclusion, our study may suggest that the ProAQT® sensor may be useful to monitor CI in patients undergoing cardiac surgery and it may provide a reliable estimate of its absolute value compared with gold standard. More studies are needed in order to validate the ProAQT® sensor and elucidate its proper use within the different clinical scenarios after cardiac surgery, as well as provide larger evidence on its use.

## Supplementary Information

**Additional file 1.** Supplementary Tables and Figures.

## Data Availability

The datasets used and analyzed during the current study are available from the corresponding author on reasonable request.

## Abbreviations

CI: Cardiac index
CVP: Central venous pressure
CPB: Cardiopulmonary bypass
SVRI: Systemic vascular resistance index
PAC: Pulmonary arterial catheter
PiCCO2: Pulse index continuous cardiac output
ICU: Intensive care unit
MAP: Mean arterial pressure
