# Supplementary material for: Study of the accuracy of a radial arterial pressure waveform cardiac output measurement device after cardiac surgery
Source: J Cardiothorac Surg. 2023 Jan 17;18:32. doi: 10.1186/s13019-023-02128-1 (PMC9843904; doi:10.1186/s13019-023-02128-1)

**Figure S1.** Flow chart of the studied patients.


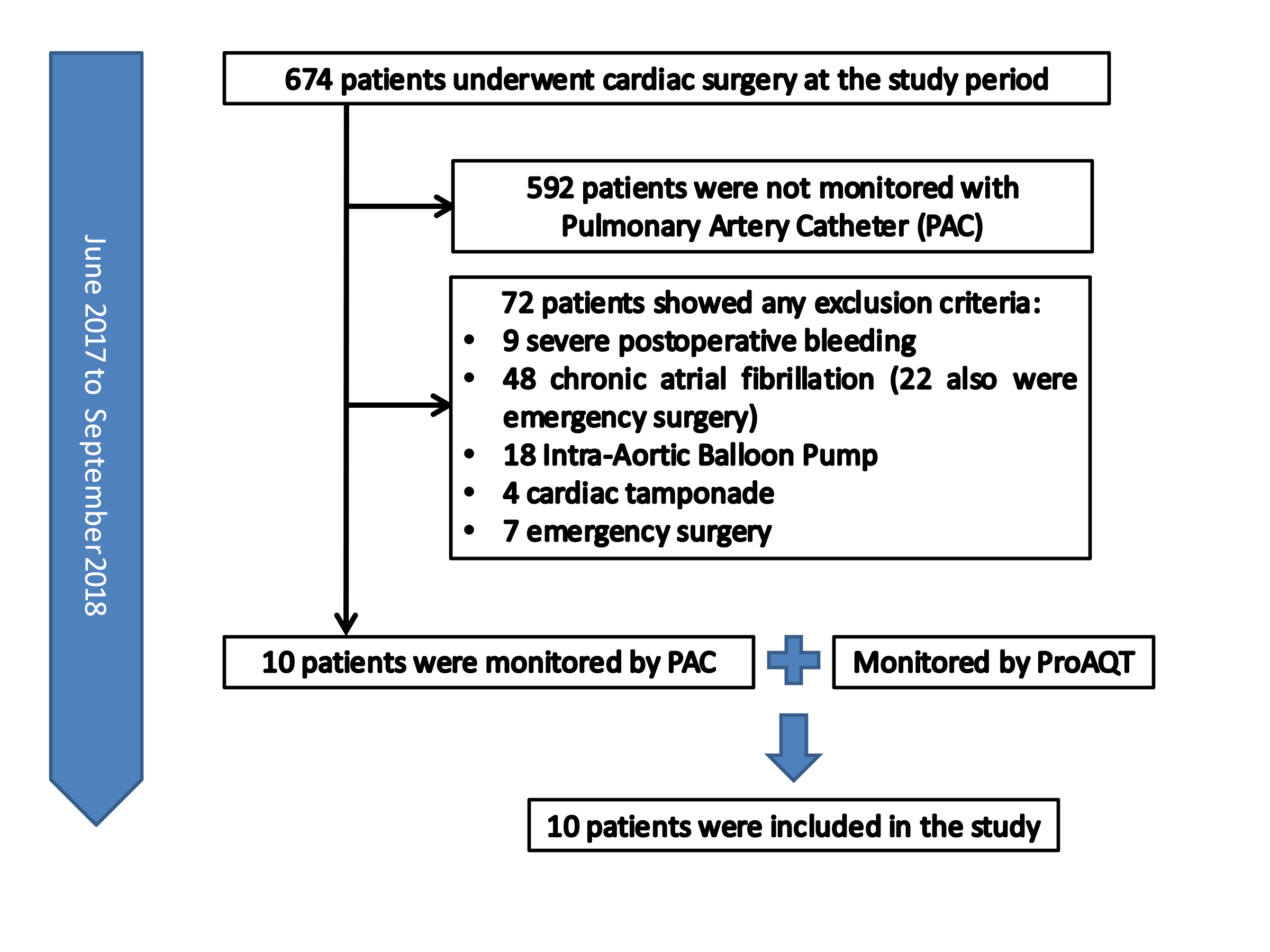


**Table S1.** Clinical characteristics and main postoperative complications of the patients include in the study.

| **Age** | **Medical past history** | **Previous**  **EF (%)** | **Surgical group** | **CPB/**  **ACC time (min)** | **Postoperative complications** | **PaO2/FiO2 ratio on ICU admission** | **Time on MV**  **(h)** |
| --- | --- | --- | --- | --- | --- | --- | --- |
| 69 | HTA, DM, DL | 43 | Aortic surgery | 145 / 95 | LCOS, AKI | 186 | 73 |
| 79 | HTA, DL | 45 | Mitral valve surgery | 118 / 79 | LCOS | 158 | 48 |
| 78 | DL | 46 | Mitral valve surgery | 102 / 87 | LCOS, pAF | 190 | 110 |
| 74 | HTA, DL | 40 | Mitral valve surgery | 125 / 94 | LCOS, pAF | 175 | 36 |
| 76 | DL | 45 | CABG (4bypass) | 145 / 91 | LCOS, AKI | 160 | 56 |
| 78 | HTA, DM, DL | 55 | Aortic surgery | 135 / 95 | LCOS, AKI, | 185 | 54 |
| 78 | HTA, DL, COPD | 50 | Mitral valve surgery | 91 / 69 | LCOS | 170 | 98 |
| 67 | HTA, DM | 35 | CABG (4bypass) | 93 / 57 | LCOS, AKI | 160 | 55 |
| 66 | HTA, DL, COPD | 35 | Mitral valve surgery | 92 / 72 | LCOS | 145 | 44 |
| 74 | HTA, DM, DL | 40 | Aortic valve surgery | 149 / 122 | LCOS | 205 | 46 |

HTA: Hypertension; DM: Diabetes mellitus; DL: Dyslipidemia; COPD: chronic obstructive pulmonary disease; EF: ejection fraction; CABG: coronary artery bypass grafting; CBP: cardiopulmonary bypass; ACC: aortic cross clamping; LCOS: Low Cardiac Output Syndrome; pAF: Paroxysmal Atrial Fibrillation; AKI: Acute Kidney Injury Type I; PaO2/FiO2: arterial partial pressure of O2 and fraction of inspired oxygen; MV: Invasive Mechanical Ventilation.

**Supplementary Content:** Degree of agreement with CI and SVRI between PAC and ProAQT® sensors across time points

Regarding SVRI measurement, there was no significant interaction between the two sensors (**Table S2**). Values for ProAQT® were significantly higher (β ̂ =0.471, p-value=0.028) and a concordance correlation coefficient of 0.17 (95% Confidence Interval: -0.08, 0.41) suggests a low concordance between the two sensors. Regarding SVRI, extreme observations for both sensors were registered at baseline for one patient, therefore an ancillary analysis was performed without this SVRI basal observation for this patient and concordance correlation coefficient increased to 0.45 (CI 95%: 0.16, 0.66), with the sensor effect being more likely to PAC (β ̂ = 0.414, p-value=0.007). **Figure S2A** shows the Bland-Altmann for SVRI (with 95% Confidence Interval) for each measurement. **Figure S2B** shows the four-quadrant plot in which the serial changes in SVRI measured with the ProAQT® were plotted against the changes in SVRI measured by PAC. The four-quadrant concordance rate, defined as the percentage of the number of data points that fall into 1 of the 2 quadrants of agreement are shown, with and without making use of an exclusion zone of 200 dyn·s^-1^·cm^-5^ based on mean SVRI. In summary, our results suggest moderate concordance rates regarding SVRI derived from ProAQT® when compared with those derived from PAC.

**Table S2.** Linear mixed model for Systemic Vascular Resistance Index.

|  | Model 1 | | | | | Model 2 | | | | |
| --- | --- | --- | --- | --- | --- | --- | --- | --- | --- | --- |
|  | Value | SE | DF | t-value | p-value | Value | SE | DF | t-value | p-value |
| (Intercept) | -0.001 | 0.272 | 86 | -0.003 | 0.998 | -0.216 | 0.338 | 79 | -0.638 | 0.525 |
| Sensor2 | 0.471 | 0.211 | 86 | 2.230 | 0.028 | 0.873 | 0.424 | 79 | 2.058 | 0.043 |
| t6 | 0.000 | 0.283 | 86 | -0.001 | 0.999 | 0.357 | 0.421 | 79 | 0.847 | 0.400 |
| t12 | -0.444 | 0.300 | 86 | -1.481 | 0.142 | -0.181 | 0.454 | 79 | -0.399 | 0.691 |
| t24 | -0.335 | 0.282 | 86 | -1.189 | 0.238 | -0.163 | 0.432 | 79 | -0.377 | 0.707 |
| t48 | -0.306 | 0.292 | 86 | -1.049 | 0.297 | -0.002 | 0.432 | 79 | -0.004 | 0.997 |
| t72 | -0.341 | 0.321 | 86 | -1.063 | 0.291 | -0.156 | 0.472 | 79 | -0.330 | 0.743 |
| t96 | -0.377 | 0.492 | 86 | -0.765 | 0.446 | -0.206 | 0.715 | 79 | -0.288 | 0.774 |
| t120 | -0.104 | 0.667 | 86 | -0.156 | 0.877 | 0.121 | 0.966 | 79 | 0.125 | 0.901 |
| Sensor2:t6 |  |  |  |  |  | -0.688 | 0.585 | 79 | -1.176 | 0.243 |
| Sensor2:t12 |  |  |  |  |  | -0.494 | 0.618 | 79 | -0.798 | 0.427 |
| Sensor2:t24 |  |  |  |  |  | -0.330 | 0.586 | 79 | -0.563 | 0.575 |
| Sensor2:t48 |  |  |  |  |  | -0.585 | 0.603 | 79 | -0.969 | 0.335 |
| Sensor2:t72 |  |  |  |  |  | -0.343 | 0.659 | 79 | -0.521 | 0.604 |
| Sensor2:t96 |  |  |  |  |  | -0.336 | 0.993 | 79 | -0.338 | 0.736 |
| Sensor2:t120 |  |  |  |  |  | -0.443 | 1.346 | 79 | -0.329 | 0.743 |

SE: Standard Error; DF: Degrees of Freedom. Sensor 2 corresponds to ProAQT® sensor.

Model 1 (Additive Model): AIC (Akaike information criterion) = 304.2, BIC (Bayesian information criterion) = 337.4, ICC (intraclass correlation coefficient) = 0.32. Model 2 (Interactive Model): AIC = 309.79, BIC = 359.34, ICC = 0.29.

**Figure S2.** Bland−Altmann plot between systemic vascular resistance index obtained with pulmonary arterial catheter and systemic vascular resistance index obtained using ProAQT® sensor (**A**). Four-quadrant plots of changes in systemic vascular resistance index measured with the ProAQT® sensor against the changes in systemic vascular resistance index measured with PAC (**B**).


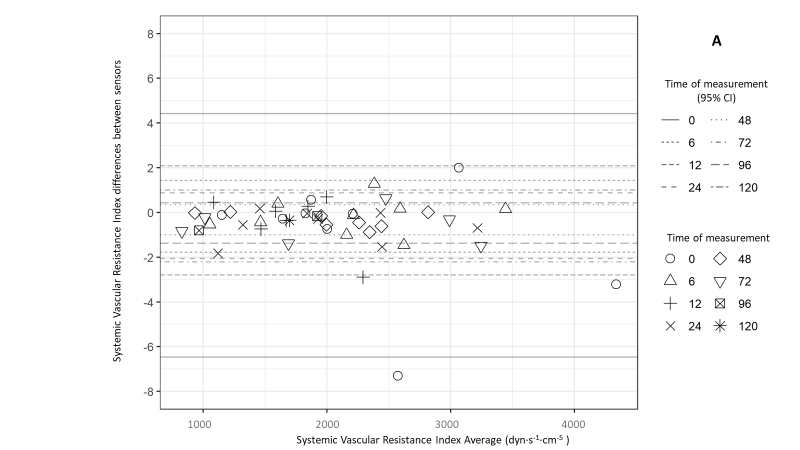


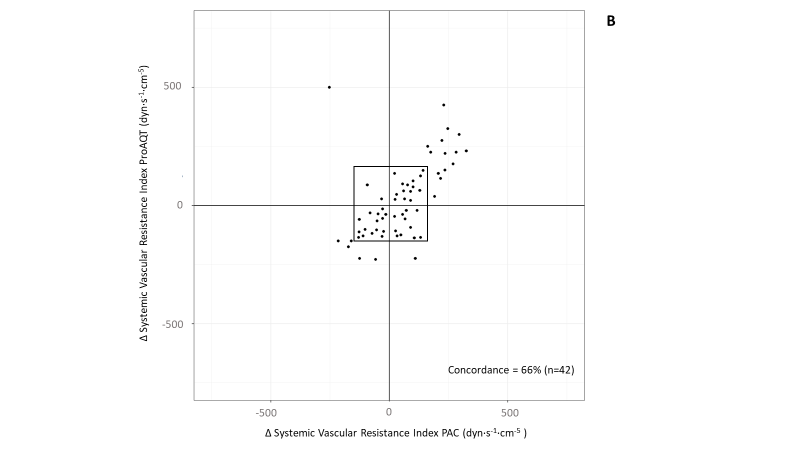

Supplement: Supplementary file 1 — Additional file 1. Supplementary Tables and Figures. [file 13019_2023_2128_MOESM1_ESM.docx]
